# Muscles Susceptibility to Ischemia-Reperfusion Injuries Depends on Fiber Type Specific Antioxidant Level

**DOI:** 10.3389/fphys.2017.00052

**Published:** 2017-02-06

**Authors:** Anne-Laure Charles, Anne-Sophie Guilbert, Max Guillot, Samy Talha, Anne Lejay, Alain Meyer, Michel Kindo, Valérie Wolff, Jamal Bouitbir, Joffrey Zoll, Bernard Geny

**Affiliations:** ^1^Equipe d'accueil 3072, Faculté de Médecine, Fédération de Médecine Translationnelle, Université de StrasbourgStrasbourg, France; ^2^Service de Physiologie et d'Explorations Fonctionnelles, Pôle de Pathologie Thoracique, Nouvel Hôpital Civil, CHRU de StrasbourgStrasbourg, France; ^3^Service de Réanimation Médico-Chirurgicale Pédiatrique Spécialisée, Hôpital de Hautepierre, CHRU de StrasbourgStrasbourg, France; ^4^Service de Réanimation Médicale, Hôpital de Hautepierre, CHRU de StrasbourgStrasbourg, France; ^5^Service de Chirurgie Cardio-Vasculaire, Pôle d'activité Médico-chirurgicale Cardiovasculaire, Nouvel Hôpital Civil, CHRU de StrasbourgStrasbourg, France; ^6^Unité neurovasculaire, Hôpital de Hautepierre, CHRU de StrasbourgStrasbourg, France; ^7^Division of Clinical Pharmacology and Toxicology, University Hospital BaselBasel, Switzerland

**Keywords:** antioxidant, ischemia-reperfusion, metabolic phenotype, mitochondria, muscle, sarcopenia, oxidative stress, peripheral arterial disease (PAD)

## Abstract

Muscle injury resulting from ischemia-reperfusion largely aggravates patient prognosis but whether and how muscle phenotype modulates ischemia-reperfusion-induced mitochondrial dysfunction remains to be investigated. We challenged the hypothesis that glycolytic muscles are more prone to ischemia-reperfusion-induced injury than oxidative skeletal muscles. We therefore determined simultaneously the effect of 3 h of ischemia induced by aortic clamping followed by 2 h of reperfusion (IR, *n* = 11) on both *gastrocnemius* and *soleus* muscles, as compared to control animals (C, *n* = 11). Further, we investigated whether tempol, an antioxidant mimicking superoxide dismutase, might compensate a reduced defense system, likely characterizing glycolytic muscles (IR-Tempol, *n* = 7). In the glycolytic *gastrocnemius* muscle, as compared to control, ischemia-reperfusion significantly decreased mitochondrial respiration (−30.28 ± 6.16%, *p* = 0.003), increased reactive oxygen species production (+79.15 ± 28.72%, *p* = 0.04), and decreased reduced glutathione (−28.19 ± 6.80%, *p* = 0.011). Less deleterious effects were observed in the oxidative *soleus* muscle (−6.44 ± 6.30%, +4.32 ± 16.84%, and −8.07 ± 10.84%, respectively), characterized by enhanced antioxidant defenses (0.63 ± 0.05 in *gastrocnemius vs*. 1.24 ± 0.08 μmol L^−1^ g^−1^ in *soleus*). Further, when previously treated with tempol, glycolytic muscle was largely protected against the deleterious effects of ischemia-reperfusion. Thus, oxidative skeletal muscles are more protected than glycolytic ones against ischemia-reperfusion, thanks to their antioxidant pool. Such pivotal data support that susceptibility to ischemia-reperfusion-induced injury differs between organs, depending on their metabolic phenotypes. This suggests a need to adapt therapeutic strategies to the specific antioxidant power of the target organ to be protected.

## Introduction

Lower limb ischemia is a frequent clinical problem resulting from trauma, hemorrhage, vascular occlusion, and/or thromboembolic events (Blaisdell, 2002; Ali et al., 2007; Beekley et al., 2008). Surgical or medical reperfusion is needed to avoid definitive necrosis, and previous studies showed that muscle irreversible damages appear likely after 4 h of ischemia (Belkin et al., 1988; Carvalho et al., 1997; Blaisdell, 2002; Martou et al., 2006; Paradis et al., 2016). After 3 h of ischemia, injury has shown to be reversible following 28 days of reperfusion. In this case however, reversibility was likely afforded by muscle regeneration (Vignaud et al., 2010; Itoh et al., 2013).

Albeit necessary, reintroduction of oxygenated blood into the ischemic areas induces unfortunately ischemia-reperfusion (IR) injury. This is significant, aggravating both local and general prognosis of the patient (Harris et al., 1986; Blaisdell, 2002; Martou et al., 2006) and supporting continuous work in order to reduce these deleterious effects.

Recently major advances have been observed in our knowledge of lower limb IR pathophysiology. Notably, interactions between muscle mitochondria and reactive oxygen species (ROS) have been emphasized (Lejay et al., 2014). Oxidative stress, which precedes mitochondrial dysfunction, arises during ischemia and is enhanced after reperfusion (Guillot et al., 2014). This suggests that modulating ROS production may reduce IR-induced injury. Accordingly, we and others demonstrated that muscle mitochondrial function is protected when ROS production is reduced and/or when the ROS produced can be efficiently handled by the antioxidant system. Such therapeutic approaches were based on both ischemic and pharmacologic conditioning (Ali et al., 2007; Makris et al., 2007; Charles et al., 2011; Tran et al., 2011; Talha et al., 2013).

Interestingly, however, interactions between mitochondria and ROS are more subtle. Significant increases in ROS production are generally considered deleterious because it induces lipid peroxidation, protein carbonylation, and mitochondrial dysfunction that is associated with reduced ATP production (Anderson and Neufer, 2006; Magder, 2006). On the other hand, a small ROS increase is a signaling pathway stimulating mitochondrial biogenesis and enhancing antioxidant defenses (Ristow, 2014; Yun and Finkel, 2014). Recently, we reported that skeletal muscles are more prone to statin-induced injury than cardiac muscles (Bouitbir et al., 2011). Metabolic phenotypes greatly differ between cardiac and skeletal muscles. Indeed, heart is highly oxidative, in association with enhanced mitochondrial respiration and antioxidant system (Masuda et al., 2003; Kowaltowski et al., 2009; Bouitbir et al., 2011).

Similar differences exist among skeletal muscles, which are classified mainly depending on their contractile and/or metabolic phenotype. Mammalian skeletal muscles are characterized by their abilities to adapt to new conditions. Indeed, changes can be detected by multiple sensors, from membrane receptors for hormones and cytokines, to metabolic sensors that detect variations in the high-energy phosphate concentration, oxygen as well as oxygen free radicals. These sensors trigger cascades of signaling pathways which may ultimately lead to changes in fiber type, especially at the level of metabolic properties (for review see Pourova et al., 2010; Schiaffino and Reggiani, 2011; Blaauw et al., 2013; Zuo et al., 2015).

Particularly, certain fibers possess a large amount of mitochondria, and rely mainly on oxidative phosphorylation. These fibers can be defined as “oxidative:” they require more oxygen and depend hence on the vascularisation to synthesize ATP. On the other hand, other fibers possess few mitochondria, and mainly rely on glycolysis to produce ATP. These fibers can be identified as “glycolytic” (Jackman and Willis, 1996; Meyer et al., 2014). Moreover, the analysis of the expression myosin heavy chains enables to differentiate four subtypes of slow (Type I) and fast twitch fibers: IIA, IIX, and IIB (Talbot and Maves, 2016). Type I fiber, such as the *soleus*, is considered as a slow oxidative fiber with high baseline respiratory rates and important antioxidant pools. Type IIA fibers use both oxidative and glycolytic metabolisms to produce ATP, and have a relatively high mitochondrial content (however lower than type I fibers). These fibers are relatively resistant to fatigue (Schiaffino, 2010). Type IIB fibers possess a low amount of mitochondria and rely mainly on glycolysis. These fibers has less antioxidant enzymes compared with type I fibers (Powers and Hamilton, 1999; Ploquin et al., 2012) and are easily fatigable, but develop tremendous amounts of strength. Type IIX fibers possess an intermediate phenotype between type IIA and type IIB fibers. Based on these characteristics, and on the fact that superficial *gastrocnemius* present with 97% of type IIb fibers and that *soleus* present with 30.6% of type I fibers (Bloemberg and Quadrilatero, 2012), we decided to study both superficial *gastrocnemius* and *soleus* in this study.

Interestingly, although Turoczi et al. described a type I muscle altered viability (Turóczi et al., 2014), other studies rather supported glycolytic muscles alterations after IR. Particularly, considering contractile force, Demirel et al. observed differences between glycolytic and oxidative muscles submitted to IR. After 4 h ischemia and 2 h reperfusion, the force-frequency curve was more altered in *extensor digitorum longus* (EDL) than in *soleus*. Further, resistance to fatigue was lower in EDL than in *soleus* (Demirel et al., 2013). Impaired fatigue resistance and contraction were also observed after IR in *gastrocnemius* (Zhang et al., 2017) and accordingly, proteins implicated in muscle contraction are known to be targets for ROS (Beckendorf and Linke, 2015), which are largely implicated in IR pathophysiology (increased lipid peroxidation and oxidized proteins; Avci et al., 2012).

Considering that oxidative skeletal muscles can also suffer from intense IR (Ali et al., 2010; Balogh et al., 2014; Turóczi et al., 2014; He and Zuo, 2015), we aimed to challenge the hypothesis their susceptibility to IR injuries might depend on their metabolic phenotypes. Particularly, oxidative skeletal muscles (as compared to glycolytic ones) should be protected from IR injuries, thanks to their antioxidant defenses.

## Materials and methods

### Ethical approval

Procedures were conducted in accordance with US National Institutes of Health guidelines. The study was approved by the institutional animal care committee of the University of Strasbourg (Comité Régional d'Ethique en Matière d'Expérimentation Animale de Strasbourg, CREMEAS, CEE35) (AL/02/09/04/08).

### Animals

We worked on adult male Wistar rats (Depré, Saint-Doulchard, France) aged 8 weeks. The temperature environment is regulated at 22 ± 2°C, and the room is submitted to a 12-h light-dark cycle. Animals had free access to food and water.

### Preoperative management

The anesthesia was induced in a hermetic cage with 4% isoflurane (Aerrane, CSP, Cournon, France) and oxygen. During the operation, the animals ventilated spontaneously, with an oxygen-delivering mask, and adapted isoflurane concentrations. The body temperature was controlled and maintained (Homeothermic blanket control unit, MINERVE, Harvard Apparatus®, Esternay, France).

### Surgical procedures and experimental design

**Figure 1:**
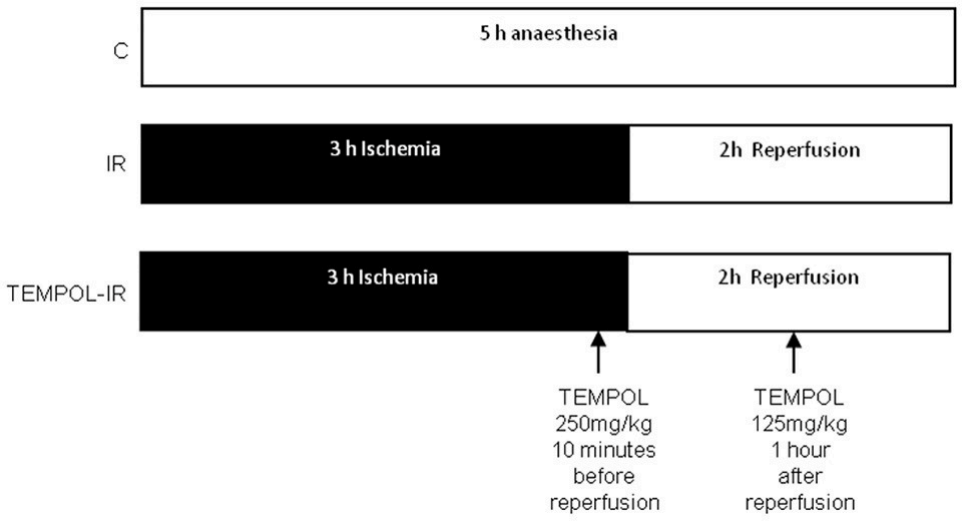
**Experimental design**. The control (C) animals (*n* = 11) underwent 5 h of general anesthesia and a sham operation. The ischemia-reperfusion (IR) animals (*n* = 11) underwent 3 h of ischemia induced by infrarenal aortic occlusion (dark bar), followed by 2 h of reperfusion (open bar). The TEMPOL-IR animals (*n* = 7) underwent the same IR protocol but 10 min before reperfusion, TEMPOL was injected (250 mg Kg^−1^) intraperitoneally (ip). A second ip injection was administered after 1 h reperfusion (125 mg Kg^−1^).

Twenty nine rats were divided in three groups (Figure 1): the control group (C, *n* = 11) underwent 5 h of general anesthesia. A midline laparotomy was performed and the abdominal aorta was exposed like in the IR groups.

The ischemia–reperfusion (IR) group (*n* = 11) underwent 3 h of ischemia induced by infra-renal aortic occlusion, and collateral vessel coagulation (GEIGER®, thermal cautery unit, Geiger Medical Technologies, Council Bluffs, Iowa, USA) followed by 2 h of reperfusion. As previously reported, ischemia was clinically characterized by cyanosis and lack of arterial pulse distal to the clamp and reperfusion was associated with recoloring and pulse return in the hind limb (Thaveau et al., 2007; Mansour et al., 2011; Pottecher et al., 2013).

Duration of ischemia was based on our and previous data demonstrating that 3 h ischemia is needed to result in significant skeletal muscle injury, which is still more severe after 6 h ischemia (Belkin et al., 1988; Thaveau et al., 2007). Chronological sequences about the cellular and molecular events occurring during skeletal muscle IR has been recently reported (Paradis et al., 2016). Interestingly, as compared to the heart which is still beating, such a long duration might be explained by the non-contracting, resting skeletal muscle generally examined. Like in the heart however (Ma et al., 2016), a no reflow occurrence might increase with long ischemia time and we therefore choose a 3 h ischemia period for our experimental protocol.

At the end of reperfusion, lactate, which is considered as a good marker of anaerobic state, was determined on total blood obtained from the tail before and after ischemia (lactate Pro device, LT710, Arkray®, KGK, Kyoto, Japan). Its decrease after opening the artery supports a successful reperfusion (Noll et al., 2012).

At the end of experiment, oxidative *soleus* and glycolytic superficial *gastrocnemius* muscles were excised (Bloemberg and Quadrilatero, 2012). For each muscle, one part was used immediately for oxidative capacities and ROS determinations. The second part was frozen in isopentane cooled by liquid nitrogen or directly in liquid nitrogen and thereafter stored for a later use.

Finally, to identify the role of oxidative stress in IR-related injury, and particularly to determine the potential protective effect of an anti-oxidant in *gastrocnemius*, a third group was given the superoxide dismutase mimetic. Rats from the Tempol-IR group (*n* = 7) had two intraperitoneal injections of 4-hydroxy-2,2,6,6-tetramethylpiperidine-1-oxyl (Tempol, Sigma-Aldrich®, St. Louis, MO, USA); 250 mg kg^−1^ at 10 min before the beginning of reperfusion and 125 mg kg^−1^ after 1 h of reperfusion (Halter et al., 2010).

### Study of muscle mitochondrial respiration

All used products were purchased from Sigma-Aldrich® (St. Louis, MO, USA).

We studied the mitochondrial respiration of skinned fibers rather than isolated mitochondria to preserve the environment and the integrity of mitochondria (Saks et al., 1998; Rasmussen and Rasmussen, 2000). The two muscles studied were superficial *gastrocnemius* and *soleus* muscles.

Oxygen consumption was measured by using a Clark-type electrode in an oxygraphic cell (Strathkelvin Instruments, Glasgow, Scotland) as previously described (Kuznetsov et al., 2008; Charles et al., 2011; Kindo et al., 2012, 2016). Maximal oxidative respiration (V_max_) was measured after adding ADP (2 mmol L^−1^) as phosphate acceptor and glutamate (5 mmol L^−1^)—malate (2 mmol L^−1^) as mitochondrial substrates. V_max_ reflects the electron flow through complexes I, III, IV, and ATP synthase.

Then, complex I was blocked with amobarbital (0.02 mmol L^−1^), and complex II was subsequently stimulated (V_succ_) with succinate (25 μmol L^−1^). The electron flow went through complexes II, III, IV, and ATP synthase.

Finally, N,N,N′,N′-tetra-methyl-p-phenylenediamine dihydrochloride (TMPD, 0.5 mmol L^−1^) and ascorbate (0.5 mmol L^−1^) were added as artificial electron donors to cytochrome c, to study complex IV activity. After the experiment, fibers were harvested and dried for 15 min at 150°C, and respiration rates were expressed as μmol O_2_ min^−1^ g^−1^ dry weight.

### Electron paramagnetic resonance

Although one method might have been sufficient, we decided to perform two complementary methods for ROS measurement in order to comfort the data. DHE staining is very useful and allows demonstrate the ROS level in tissue and EPR is viewed as an excellent technique, measuring the ROS production by the tissue (Dikalov et al., 2007; Kuznetsov et al., 2011; Lejay et al., 2015).

Electron Paramagnetic Resonance (EPR) allows determining superoxide (${\text{O}}_{2}^{-}$) formation.

${\text{O}}_{2}^{.-}$ concentration was determined with a specific spin probe 1-hydroxy-3-methoxycarbonyl-2, 2, 5, 5-tetramethylpyrrolidine hydrochloride (CMH, Noxygen®, Elsach, Germany), oxidized by impaired electron (Dikalov et al., 2007; Lejay et al., 2015; Kindo et al., 2016).

All used substrates for the Krebs Hepes buffer were purchased from Sigma-Aldrich® (St. Louis, MO, USA).

Ten 2-mm pieces of muscles were cut and washed twice with the Krebs Hepes Buffer (NaCl 99 mmol L^−1^; KCl 4.69 mmol L^−1^; CaCl_2_ 2.5 mmol L^−1^; MgSO_4_ 1.2 mmol L^−1^; NaHCO_3_ 25 mmol L^−1^; KH_2_PO_4_ 1.03 mmol L^−1^; D(+)Glucose 5.6 mmol L^−1^; Na-hepes 20 mmol L^−1^; pH 7.4) containing 25 μmol L^−1^ deferoxamine and 5 μmol L^−1^ diethyldithiocarbamate (DETC) to minimize CMH auto-oxidation. Then, they were incubated in a plate at 37°C with the spin probe CMH (200 μmol L^−1^) for 30 min under controlled pressure (20 mmHg) and gas mix (N_2_: 97.8%, O_2_: 2.8%) to mimic tissular environment (Noxygen®, Elsach, Germany). The incubation of tissues is stopped by placing the plate on ice. Forty-one microliters of the supernatant are injected in a disposable capillary tube, and placed inside the cavity of the e-scan spectrometer (Bruker Win-EPR®, Elsach, Germany) for data acquisition at 15°C (The used EPR settings: Centre Field 3461.144 g, microwave power 21.85 mW, modulation amplitude 2.40 G, sweep time 5.24 s (10 scans), sweep width 60 G, number of lag curve points 1).

The amplitude of the signal is measured, and the concentration of CM-radical is determined by calibration with standard concentrations of the radicals CM. After the EPR measurement, pieces of muscles are dried for 15 min at 150°C, and ${\text{O}}_{2}^{.-}$ production was expressed in μmol min^−1^ mg^−1^ dry weight.

### Dihydroethidium staining

Dihydroethidium staining (DHE, Sigma-Aldrich®) allows detecting the presence of ROS in skeletal muscles.

Frozen muscles were cut on serial sections (10 μm-thicks) with a cryostat microtome and thaw mounted on glass slides and incubated with DHE (2.5 μmol L^−1^). DHE produced red fluorescence when oxidized to ethidium bromide (EtBr) by ${\text{O}}_{2}^{.-}$ (Mulsch et al., 2001; Talha et al., 2013; Guillot et al., 2014). As compared to other markers, the interest of DHE is its relatively good specificity to ${\text{O}}_{2}^{.-}$.

After staining, sections were examined under an epifluorescence microscope (Eclipse E800, Nikon, New York, USA) with a 20X epifluorescence objective. The emission signal was recorded with a Zeiss 573–637 nm filter. We analyzed micrographs with Adobe Photoshop 6.0 (USA). The results were reported in percentage of control group.

### Measurement of reduced glutathione

The glutathione peroxidase is an important intracellular antioxidant, because it is the primary enzyme responsible for H_2_O_2_ detoxification in mitochondria (Antunes et al., 2002; Zoccarato et al., 2004).

All used products were purchased from Sigma-Aldrich® (St. Louis, MO, USA).

Reduced glutathione (GSH) was measured in the different muscles by monitoring the reduction of 5,5′-dithio-bis-(2-nitrobenzoic acid) (DTNB) to 5-thio-2-nitrobenzoate (TNB) in presence of GSH at 412 nm (Akerboom and Sies, 1981; Bouitbir et al., 2011). Briefly, to determine GSH content, tissue samples were mixed with cold 5% 5-sulfosalicylic acid, after 10 min of incubation on ice, the mixture was centrifuged at 4000 rpm for 10 min at 4°C to remove proteins. The samples were then diluted in cold MOPS buffer, at room temperature. The diluted sample was added to glutathione reductase (24 units mL^−1^) and MOPS (0.1 mol L^−1^) containing 1 mmol L^−1^ EDTA, 0.3 mmol L^−1^ NADPH, and 0.22 mmol L^−1^ DTNB. The absorbance was continuously monitored for 1 min, as an index of DTNB reduction by GSH contained in the sample. Reduced glutathione was expressed in μmol L^−1^ g^−1^.

### Statistical analysis

Values are expressed as mean ± SEM. n indicates the number of animals. Statistical analyses were performed with Student's *t*-test for unpaired data. When the variances were significantly different, we used the Student's unpaired *t*-test with Welch's correction. For the lactate rates comparison statistical significance was measured by ANOVA followed by the post-test Newman Keuls (GraphPad Prism 5, Graph Pad Software, Inc., San Diego, CA, USA). Values of *p* < 0.05 were considered to be statistically significant.

## Results

### Ischemia significantly increased blood lactates, which returned to baseline values after reperfusion (Figure 2)

Plasma lactate kinetic demonstrated the efficiency of IR. After 3 h ischemia, lactate increased significantly (10.9 ± 2.0 *vs*. 2.2 ± 0.2 mmol L^−1^, +395.4%; *p* < 0.001). Then, at reperfusion, lactate decreased significantly (3.1 ± 0.5 *vs*. 10.9 ± 2.0 mmol L^−1^, *p* < 0.001) and there was no significant difference in lactate values between before ischemia and after reperfusion (2.2 ± 0.2 *vs*. 3.1 ± 0.5 mmol L^−1^, NS, respectively).

**Figure 2:**
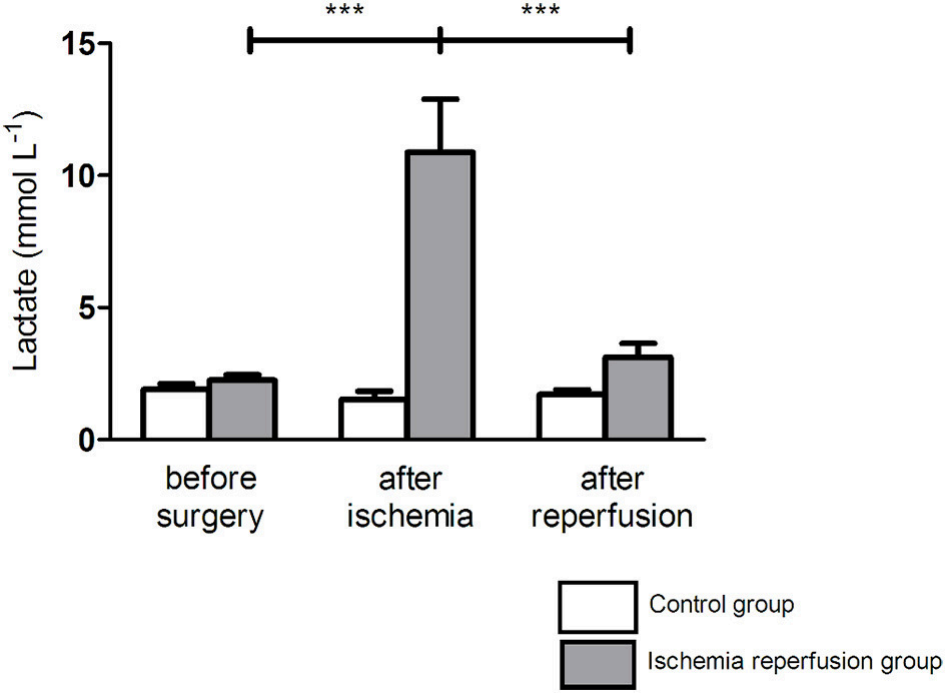
**Lactate concentrations increased with ischemia and returned to baseline level after reperfusion**. Ischemia-reperfusion (IR) group (gray bars), control group (white bars). Lactates levels were measured before and after 3 h of ischemia, and after 2 h of reperfusion. Results were expressed as mean ± SEM. ^***^p < 0.001 compared to after ischemia.

### IR impaired mitochondrial respiration, increased ROS production, and reduced antioxidant defenses in glycolytic *gastrocnemius* muscles (Figure 3)

#### Mitochondrial respiratory chain complex activities (Figure 3A)

In *gastrocnemius*, maximal respiration rate (V_max_), reflecting the activity of complexes I, III, IV, and V, was significantly altered after IR as compared to the control group (4.51 ± 0.44 *vs*. 6.61 ± 0.42 μmol O_2_ min^−1^ g^−1^ dry weight, respectively, *p* < 0.05).

**Figure 3:**
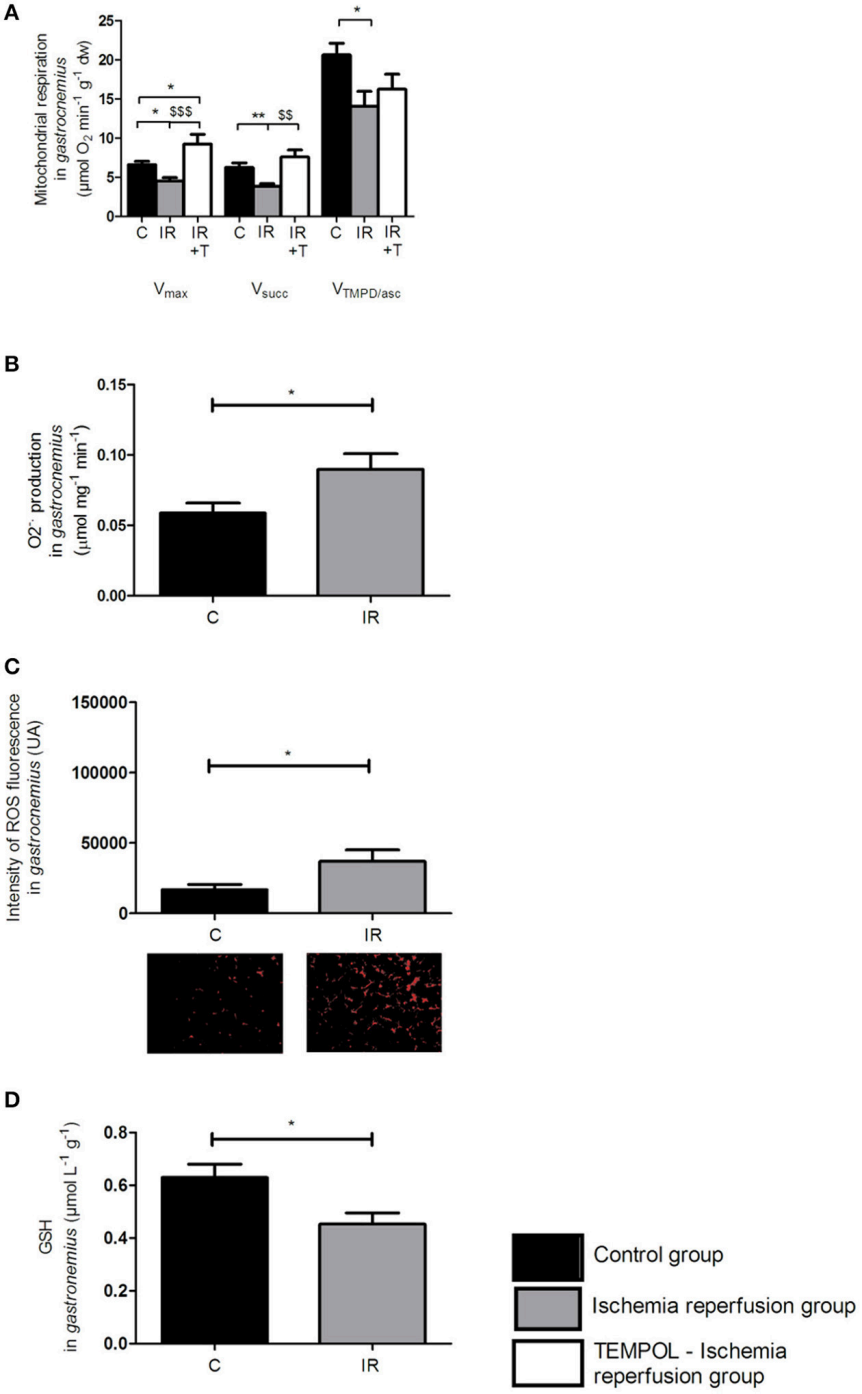
**Ischemia-reperfusion impaired glycolytic ***gastrocnemius*** muscle, and an increase of antioxidant protected this muscle against an IR injury. (A)** Mitochondrial respiration in the *gastrocnemius*. **(B,C)** ROS production in the *gastrocnemius:* superoxide anion production measured by EPR with a specific probe CMH **(B)** and intensity of fluorescence labeled with oxidative dye DHE **(C)**. **(D)** Reduced glutathione in the *gastrocnemius*. The black bars represent the C group (control group) and the gray bars represent the IR group (3 h of ischemia and 2 h of reperfusion). V_max_: complexes I, III, IV, V activities; Vsucc: complexes II, III, IV, V activities; V_TMPD/asc_: complex IV activity. Results were expressed as mean ± SEM. ^*^p < 0.05, ^**^p < 0.01 compared to the control group. ^$$^*p* < 0.01, ^$$$^*p* < 0.001 compared to the IR group. C, Control group; IR, Ischemia-reperfusion group. IR+T: TEMPOL-IR group.

Similarly, V_succ_, reflecting the activity of complexes II, III, IV, and V, was decreased after IR (3.82 ± 0.36 *vs*. 6.23 ± 0.61 μmol O_2_ min^−1^g^−1^ dry weight, respectively, *p* < 0.01).

Finally, V_TMPD/Asc_, reflecting the complex IV activity, was decreased after IR (14.05 ± 1.92 *vs*. 20.61 ± 1.49 μmol O_2_ min^−1^ g^−1^ dry weight compared to the control group, respectively, *p* < 0.05).

Thus, IR impaired all complexes of the mitochondrial respiratory chain of the *gastrocnemius* muscle.

#### ROS production (Figures 3B,C)

The graph B represents the ${\text{O}}_{2}^{.-}$ production measured by EPR, demonstrating that ${\text{O}}_{2}^{-}$ production was significantly increased compared to control group (0.09 ± 0.01 *vs*. 0.06 ± 0.01 μmol mg^−1^ min^−1^, respectively, *p* = 0.037) in the *gastrocnemius*.

This result was confirmed in the graph C representing DHE staining. Indeed, ROS content was increased after IR as compared to the control group (36,860 ± 8127 *vs*. 16,720 ± 3674 UA, respectively, *p* = 0.04).

#### Antioxidant defenses (Figure 3D)

The reduced glutathione produced in *gastrocnemius* was critically decreased after IR as compared to the control group (0.63 ± 0.05 *vs*. 0.45 ± 0.04 μmol L^−1^ g^−1^, *p* = 0.024, respectively).

### Effects of IR on the oxidative *soleus* muscle (Figure 4)

#### Mitochondrial respiratory chain complex activities (Figure 4A)

Less-IR induced damage is observed in oxidative muscle. In *soleus*, the maximal respiration rate (V_max_), although showing a slight tendency to decrease, was not significantly altered after IR as compared to the control group (8.94 ± 0.60 *vs*. 9.56 ± 0.58 μmol O_2_ min^−1^ g^−1^ dry weight, respectively, *p* = 0.469).

**Figure 4:**
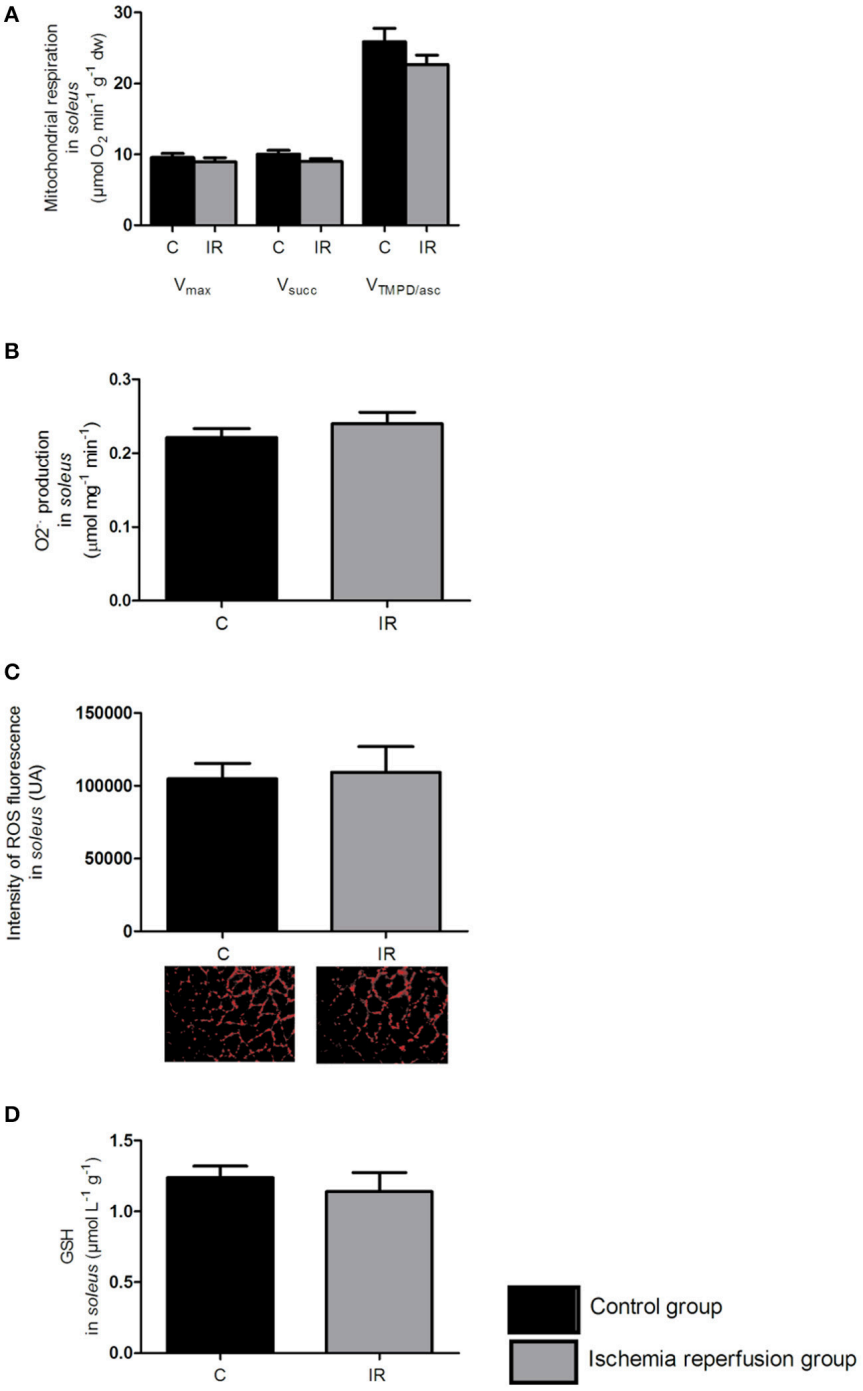
**Ischemia-reperfusion effects on the oxidative ***soleus*** muscle. (A)** Mitochondrial respiration in the *soleus*. **(B,C)** ROS production in the *soleus:* superoxide anion production measured by EPR with a specific probe CMH **(B)** and intensity of fluorescence labeled with oxidative dye DHE **(C)**. **(D)** Reduced glutathione in the *soleus*. The black bars represent the C group (control group) and the gray bars represent the IR group (3 h of ischemia and 2 h of reperfusion). V_max_: complexes I, III, IV, V activities; Vsucc: complexes II, III, IV, V activities; V_TMPD/asc_: complex IV activity. Results were expressed as mean ± SEM. C, Control group, IR, Ischemia-reperfusion group.

Similarly, V_succ_, was not significantly modified after IR (8.99 ± 0.43 *vs*. 10.05 ± 0.52 μmol O_2_ min^−1^ g^−1^ dry weight, respectively, *p* = 0.135).

V_TMPD/Asc_, showed the same profile and was not significantly altered after IR, as compared to the control group (22.70 ± 1.32 *vs*. 25.88 ± 1.92 μmol O_2_ min^−1^g^−1^ dry weight, respectively, *p* = 0.187).

#### ROS production (Figures 4B,C)

Concerning the oxidative *soleus*, the graph B shows that ${\text{O}}_{2}^{.-}$ production was not modified as compared to the control group (0.24 ± 0.01 *vs*. 0.22 ± 0.01 μmol mg^−1^ min^−1^, respectively, *p* = 0.36).

Further, the graph C confirmed that ROS content remained unchanged after IR as compared to the control group (109,300 ± 17,650 *vs*. 104,800 ± 10,640 UA, *p* = 0.83, respectively).

#### Antioxidant defenses (Figure 4D)

In the *soleus*, IR did not altered the reduced glutathione content (1.14 ± 0.13 *vs*. 1.24 ± 0.08 μmol L^−1^ g^−1^, *p* = 0.54, as compared to the control group).

Taken together, these data demonstrate that unlike the glycolytic muscle *gastrocnemius*, the oxidative *soleus* is well-protected from IR-induced deleterious effects.

**Figure 5:**
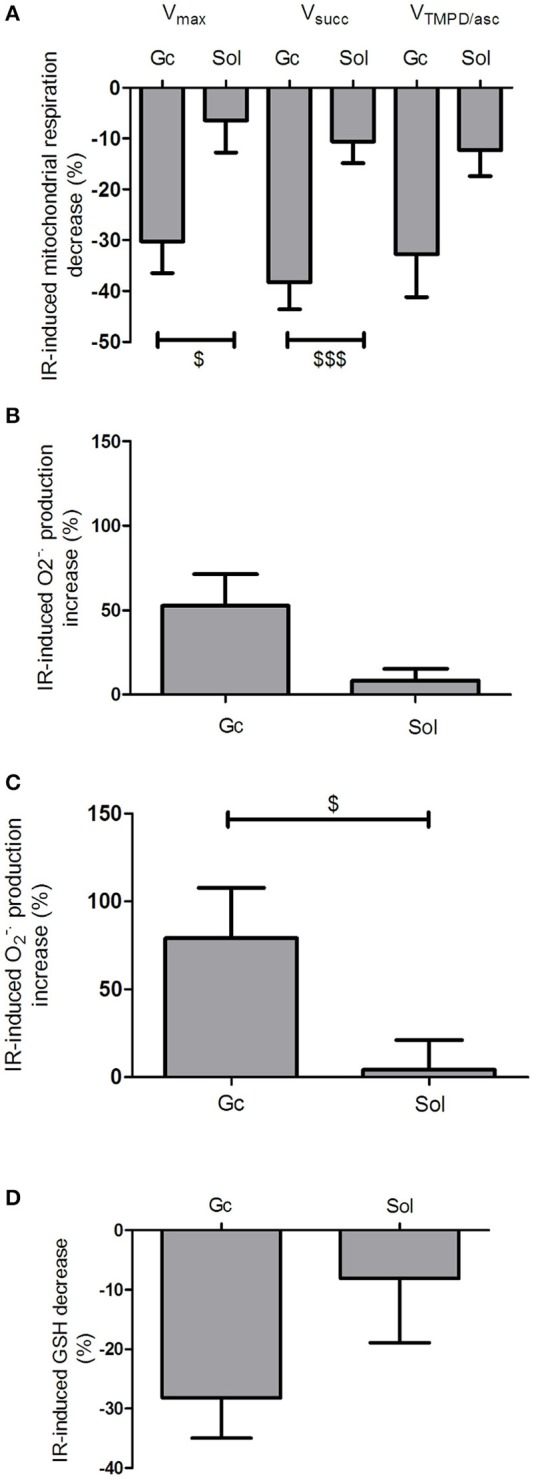
**Ischemia reperfusion altered specifically glycolytic compared to oxidative skeletal muscles. (A)** IR-induced mitochondrial respiration in the *gastrocnemius* (Gc) compared to the *soleus* (Sol) expressed in %. **(B,C)** ROS production in the *gastrocnemius* (Gc) compared to the *soleus* (Sol) expressed in %. Superoxide anion production measured by EPR with a specific probe CMH **(B)** and intensity of fluorescence labeled with oxidative dye DHE **(C)**. **(D)** The use of IR-induced reduced glutathione was compared in *gastrocnemius* (Gc) and *soleus* (Sol), and expressed in %. Results were expressed as mean ± SEM. ^$^*p* < 0.05, ^$$$^*p* < 0.001 compared to *gastrocnemius*.

Thus, the alteration of V_max_ was markedly greater in *gastrocnemius* than in *soleus* (−30.28 ± 6.17 *vs*. −6.44 ± 6.30%, respectively, *p* = 0.014). Similarly, the alteration of V_succ_ and of V_TMPD/Asc_ were largely enhanced in *gastrocnemius* compared to *soleus* (−38.28 ± 5.31 *vs*. −10.54 ± 4.30%, respectively, *p* = 0.0006) and (−32.71 ± 8.48 *vs*. −12.29 ± 5.08%, *p* = 0.052), respectively (Figure 5A).

Such lack of oxidative skeletal muscle alteration was associated with an absence of oxidative stress.

Accordingly, ROS production increased more in *gastrocnemius* than in *soleus* (+39.80 ± 16.32 *vs*. +8.36 ± 6.99% in *gastrocnemius* and *soleus*, respectively, *p* = 0.088, Figure 5B).

Similar data were obtained when using DHE staining (+79.15 ± 28.72 *vs*. +4.32 ± 16.84% of alteration in *gastrocnemius* and *soleus*, respectively, *p* = 0.034, Figure 5C).

Finally, the antioxidant defenses were reduced by IR only in the *gastrocnemius* as compared to the *soleus* (−28.20 ± 6.80 *vs*. −8.07 ± 10.84%, respectively, *p* = 0.148, Figure 5D).

### Metabolic phenotypes implications in muscle susceptibility to IR

The metabolic phenotype differs between *gastrocnemius* and *soleus* muscles and might therefore explain their specific sensitivity to IR.

Indeed, as compared to oxidative ones, glycolytic muscles are characterized by lower mitochondrial respiratory chain complex activities. This holds true for all complexes. Thus, V_max_, V_succ_, and V_TMPD/Asc_ are smaller in *gastrocnemius* as compared to *soleus* (6.6 ± 0.4 *vs*. 9.6 ± 0.6; 6.2 ± 0.6 *vs*. 10.0 ± 0.5; and 20.6 ± 1.5 *vs*. 25.9 ± 1.9 μmol O_2_ min^−1^ g^−1^ dry weight, respectively, *p* < 0.001) (Figures 3A, 4A).

Further, ${\text{O}}_{2}^{.-}$ production determined by EPR was significantly lower in *gastrocnemius* than in *soleus* (0.059 ± 0.007, 0.221 ± 0.012 μmol mg^−1^ min^−1^, respectively, *p* < 0.001, Figures 3B, 4B). This is confirmed by DHE staining demonstrating that ROS level was a six-fold lower in *gastrocnemius* as compared to *soleus* (16,720 ± 3674 *vs*. 104,800 ± 10,640 UA, *p* < 0.001, respectively, Figures 3C, 4C).

Finally, *gastrocnemius* muscle antioxidant defenses, as inferred from GSH are two-fold lower in *gastrocnemius* compared to *soleus* (0.63 ± 0.05 *vs*. 1.24 ± 0.08 μmol L^−1^ g^−1^, *p* < 0.0001, respectively, Figures 3D, 4D).

In view of these data we tested the hypothesis that the reduced antioxidant defenses of *gastrocnemius* muscle might be overcome by administration of tempol, an efficient antioxidant, and therefore confer protection against IR to this glycolytic muscle.

With tempol, mitochondrial oxidative capacities were no longer impaired by IR in *gastrocnemius* (Figure 3A).

Thus, in the Tempol-IR group, V_max_ was not impaired by IR (9.25 ± 1.25, *vs*. 3.82 ± 0.36 μmol O_2_ min^−1^g^−1^ dry weight, *p* < 0.001 and 6.23 ± 0.61 μmol O_2_ min^−1^g^−1^ dry weight, *p* < 0.05, for IR and control group, respectively).

Similarly, V_succ_ was not impaired by IR (7.58 ± 0.90 *vs*. 3.82 ± 0.36 μmol O_2_ min^−1^g^−1^ dry weight, *p* < 0.01, and 6.23 ± 0.61 μmol O_2_ min^−1^ g^−1^ dry weight, *p* = NS for IR and control group, respectively). V_TMPD/asc_ (16.24 ± 1.92) was not significantly different from IR and control values (14.05 ± 1.92 and 20.61 ± 1.49 μmol O_2_ min^−1^ g^−1^ dry weight, for IR and control group, respectively).

## Discussion

The main result of this study is that skeletal muscle metabolic phenotype importantly modulates deleterious effects of lower limb IR. Thus, as compared to the glycolytic *gastrocnemius*, the oxidative *soleus* appeared protected and demonstrated no significant mitochondrial dysfunction. In *soleus*, higher anti-oxidant capacity likely participates in such protection since oxidative stress is a key factor inducing muscle impairment during IR. Accordingly, when treated with an antioxidant, *gastrocnemius* muscle acted as *soleus*, showing no more significant alteration although submitted to the same IR protocol.

Vascular ischemia is very common and corresponds to a public health issue both because of its increasing occurrence and its very bad prognosis (Yassin et al., 2002). Thus, major amputation is still needed in 25–40% of cases, and the risk of death is as high as 25% after the first clinical sign of peripheral arteriopathy (Norgren et al., 2007; Taylor et al., 2009; Ryan et al., 2015). Interestingly, muscle alteration significantly impacts both local and general prognosis. Thus, rhabdomyolysis is well-known to participate in remote organ alteration including kidneys, the heart and lungs (Fowkes et al., 2006; Ali et al., 2007; McMahon et al., 2013).

Although some therapeutic strategies have been shown effective, results are not always consistent, raising the issue of a specific organ dependency to IR (Mansour et al., 2014). Concerning skeletal muscles, metabolic phenotype is a good candidate since degrees of fiber injury vary among contractile muscle phenotypes (Chan et al., 2004). Woitaske et al. observed an increased mitochondrial swelling and disrupted sarcoplasmic reticulum in the glycolytic EDL as compared to *soleus* muscle (Woitaske and McCarter, 1998). Accordingly, IR- induced contractile dysfunction was recently shown to be reduced in *soleus* as compared to EDL (Demirel et al., 2013).

Based on different muscle phenotypes, several mechanisms might explain such potential increased sensitivity of glycolytic muscle to IR injury. Particularly, mitochondrial content and redox balance deserve discussion.

Mitochondrial density is about two-fold greater in oxidative than in glycolytic muscles (Jackman and Willis, 1996; Picard et al., 2008), likely participating in the enhanced mitochondrial respiration observed in *soleus* as compared to *gastrocnemius* muscles (Anderson and Neufer, 2006; Meyer et al., 2014). Recently, induction of mitochondrial biogenesis has been shown to protect against apoptosis in L6 myoblats (Dam et al., 2013), and we observed that decrease in PGC1 beta, which likely participate in mitochondrial biogenesis, resulted in a reduced adaptation of muscles to cope with high energy demands (Gali Ramamoorthy et al., 2015). Such relationship between the number of mitochondria and muscle responses to stress likely apply in the setting of IR. Accordingly, Flück et al. demonstrated that IR-induced skeletal muscle rhabdomyolysis is a fiber type-specific phenomenon, modulated by mitochondria reserves (Fluck et al., 2015).

However, besides mitochondrial respiration *per-se*, redox status appears to be a key factor in muscle functioning (for review see Zuo and Pannell, 2015). Even during physiological contraction (eccentric and/or concentric), muscle mitochondria generate large amounts of ROS, that might allow either muscular adaptation or muscular damage. Thus, depending likely on ROS levels resulting from the pro-/antioxidant balance, consequences might be good (mitochondrial biogenesis, antioxidant pathway stimulation, enhanced glucose uptake), bad (mitochondrial dysfunctions, reduced ATP synthesis) or even ugly (mPTP opening and apoptosis) (Lejay et al., 2014; Zuo et al., 2015).

In this view, exercise is interesting to consider. Indeed, exercise-related beneficial effects are largely reduced when exercise-induced ROS signaling is decreased by enzymatic inhibitors or antioxidant supplementation (Meier et al., 2013; Strobel et al., 2014; Merry and Ristow, 2016). Further, the effects of exercise likely depend on muscle metabolic phenotype. For instance, exercise has a stronger influence on red muscle phenotype, as compared to white muscle in salmonids (Morash et al., 2014). Similarly, uphill-, as compared to down-hill training enhanced mitochondrial function specifically in both *soleus* and *vastus intermedius* skeletal muscle (Schlagowski et al., 2016).

Increased antioxidant defenses also characterize oxidative muscles. This likely explains why statins optimize cardiac mitochondria, but impairs skeletal muscle function by inducing different levels of ROS (Bouitbir et al., 2011, 2015). Concerning skeletal muscles, in agreement with previous reports (Anderson and Neufer, 2006; Picard et al., 2012), we observed greater glutathione peroxidase activity in the *soleus* than in the superficial *gastrocnemius* This support that the oxidative skeletal muscle may possess a greater capacity to scavenge ROS (Picard et al., 2012). Since enhanced ROS production occur early and participate significantly in IR deleterious effects (Tran et al., 2011; Guillot et al., 2014; Lejay et al., 2014), the fact that oxidative muscles naturally equip with higher antioxidant contents than glycolytic muscles likely protect them in the setting of IR. Further, an adaptation related to oxidative resistance might have been triggered during IR. Such a possibility, likely working in the setting of preconditioning, can perhaps not be totally developed in the relatively short time frame of our study but, one might suggest that the antioxidant pool located in the blood might also have been mobilized.

**Figure 6:**
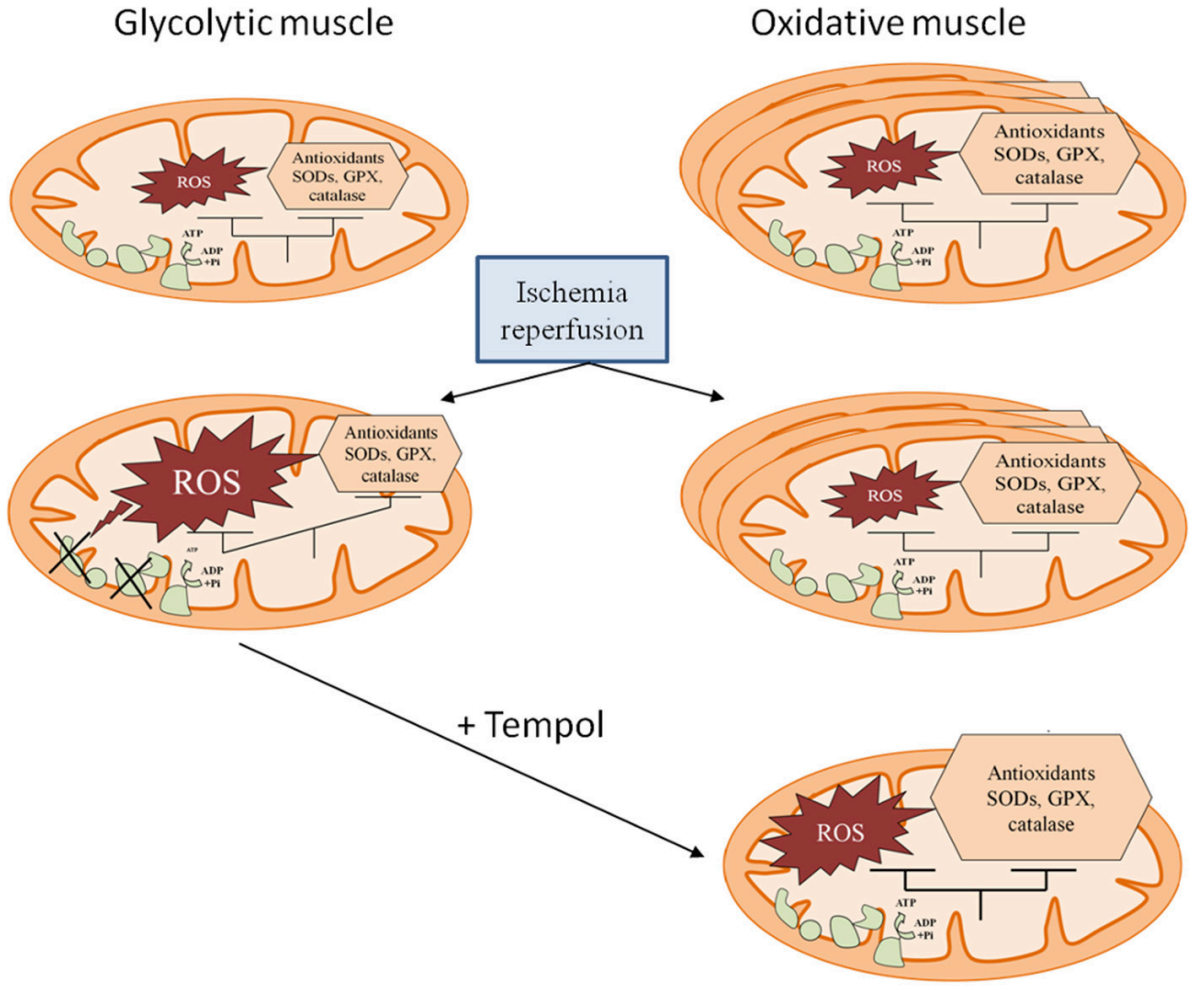
**Schema illustrating skeletal muscle susceptibility to ischemia-reperfusion, depending on metabolic phenotype**. Oxidative skeletal muscles are characterized by increased mitochondrial content and enhanced antioxidant system allowing better protection against ischemia-reperfusion supported by maintained mitochondrial respiration and low reactive oxygen species (ROS) production. Antioxidant adjunction protects glycolytic muscle, further demonstrating the key role of oxidative stress handling and therefore muscle metabolic phenotype. SODs, Superoxide dismutases; GPX, glutathione peroxidase.

To further test that enhanced antioxidant defenses allow oxidative skeletal muscles being protected against IR, we determined whether the glycolytic muscle *gastrocnemius* might be protected against IR when its antioxidant defense is improved. Accordingly, tempol therapy significantly attenuated the deleterious effects of IR. Such data are in agreement with a previous work (Tran et al., 2011) and further suggest a key role of the antioxidant defenses and likely explain -at least partly- the specific muscle susceptibility to IR (Figure 6).

As perspectives, further studies should be able to demonstrate that ROS staining be mainly colocalized within type II fibers during IR. It might be useful to determine the molecular mechanisms involved in muscle protection, signaling ranging from mitochondrial biogenesis to atrophy-hypertrophy pathways. Besides ROS-activated signaling pathways such as MAPK, PI3K/Akt signaling, PKC, and inflammation signaling (Görlach et al., 2015), a special insight should be directed toward the SAFE and RISK pathways which modified interplay might account for increased susceptibility of diabetic muscles to ischemia (Lejay et al., 2016).

## Conclusion

This study supports that glycolytic muscles are more prone to IR than oxidative ones. Such muscle susceptibility appears linked to muscle metabolic phenotypes, involving mainly muscle mitochondrial oxidative capacity and antioxidant defenses. This might explain difficulties to convert experimental data well-focused on specific muscle fibers toward clinical setting, since human muscles show more phenotypic heterogeneity. More widely, these data suggest that therapeutic approaches should be adapted to target organ redox capacities (potentially modified by comorbidities), and that mechanisms increasing either mitochondrial function and/or antioxidant defense should allow better protection.

## Author contributions

Conception or design of the work: AC, AG, MG, ST, AL, JB, BG. Acquisition, analysis: AC, AG, MG, ST, AL, JB, JZ. Interpretation of data for the work: AC, AG, AM, MK, VW, JZ, BG. Drafting or revising the work: AC, AG, MG, VW, JB, JZ, BG. Final approval: AC, AG, MG, ST, AL, AM, MK, VW, JB, JZ, BG. Agreement to be accountable of all aspects of the work: AC, AG, MG, ST, AL, AM, MK,VW, JB, JZ, BG.

### Conflict of interest statement

The authors declare that the research was conducted in the absence of any commercial or financial relationships that could be construed as a potential conflict of interest. The reviewer ZL and handling Editor declared their shared affiliation, and the handling Editor states that the process nevertheless met the standards of a fair and objective review.
